# ‘Valves’ of the angular vein: Orbicularis oculi, depressor supercilii, and zygomaticus minor

**DOI:** 10.1371/journal.pone.0276121

**Published:** 2022-10-13

**Authors:** Joe Iwanaga, R. Shane Tubbs, Hongtae Kim, Mi-Sun Hur

**Affiliations:** 1 Department of Oral and Maxillofacial Anatomy, Graduate School of Medical and Dental Sciences, Tokyo Medical and Dental University, Tokyo, Japan; 2 Department of Neurosurgery, Tulane Center for Clinical Neurosciences, Tulane University School of Medicine, New Orleans, LA, United States of America; 3 Department of Neurology, Tulane Center for Clinical Neurosciences, Tulane University School of Medicine, New Orleans, LA, United States of America; 4 Dental and Oral Medical Center, Kurume University School of Medicine, Kurume, Fukuoka, Japan; 5 Division of Gross and Clinical Anatomy, Department of Anatomy, Kurume University School of Medicine, Kurume, Fukuoka Japan; 6 Department of Anatomical Sciences, St. George’s University, St. George’s, Grenada, West Indies; 7 Department of Structural & Cellular Biology, Tulane University School of Medicine, New Orleans, LA, United States of America; 8 Department of Surgery, Tulane University School of Medicine, New Orleans, LA, United States of America; 9 Department of Neurosurgery and Ochsner Neuroscience Institute, Ochsner Health System, New Orleans, LA, United States of America; 10 University of Queensland, Brisbane, Australia; 11 Department of Anatomy, Daegu Catholic University School of Medicine, Daegu, Korea; Liverpool John Moores University, UNITED KINGDOM

## Abstract

**Objectives:**

The aim of this study was to elucidate the positional relationship between the courses of the angular veins and the facial muscles, and the possible roles of the latter as alternative venous valves.

**Methods:**

The angular veins of 44 specimens of embalmed Korean adult cadavers were examined. Facial muscles were studied to establish their relationships with the angular vein, including the orbicularis oculi (OOc), depressor supercilii (DS), zygomaticus minor (Zmi), zygomaticus major (Zmj), and levator labii superioris (LLS).

**Results:**

In the upper face of all specimens, the angular vein passed through the DS and descended to the medial palpebral ligament. In the midface, it passed between the origin of the levator labii superioris alaeque nasi (LLSAN) and the inferior OOc fibers. The vein coursed along the deep surface of the inferior margin of the OOc in all specimens. At the level of the nasal ala, the course of the angular vein was classified into three types: in type I it passed between the LLS and Zmi (38.6%), in type II it passed between the superficial and deep fibers of the Zmi (47.7%), and in type III it passed between the Zmi and Zmj (13.6%). In the lower face of all specimens, the angular or facial vein passed through the anterior lobe of the buccal fat pad.

**Conclusion:**

This study found that the angular vein coursed along the sites where facial muscle contractions are assumed to efficiently compress the veins, likely controlling venous flow as valves. The observations made and analysis performed in this study will improve the understanding of the physiological function of the facial muscles as alternative venous valves.

## Introduction

The angular vein, or upper part of the facial vein, is formed by the junction of the supratrochlear and supraorbital veins at the root of the nose and becomes the facial vein near to the level of the zygomaticus major (Zmj) [1]. As the angular vein lies beside the nose, it receives small veins from the nose and both eyelids [2]. After it joins the superior labial vein it becomes the facial vein, which passes under the Zmj, risorius, and platysma, and descends to the anterior border of the masseter [3].

Several textbooks indicate that the angular, facial, and ophthalmic veins lack valves [4–6], but some authors have asserted that the superior ophthalmic and facial veins have valves [7, 8]. Nishihara et al. (1995) [7] and Zhang and Stringer (2010) [8] reported finding no valves in the angular vein but many in its tributaries. Thus, there is currently no consensus on whether the venous system in the face has valves or can control blood flow in some other way.

Skeletal muscle contraction is well known to be important in controlling venous blood flow in the lower limbs and in controlling tears in the lacrimal sac and gland. During standing, venous return from the lower limbs is highly dependent on muscular activity, especially calf and foot muscle contractions, which is known as the ‘muscle pump’ [3, 9, 10]. When the posterior leg muscles contract, blood is proximally pumped into the deep veins [3, 11]. In the facial region, the palpebral portion of the orbicularis oculi (OOc) dilates the lacrimal sac, thereby facilitating tear aspiration. The lacrimal portion of the OOc likely compresses the lacrimal sac, forcing tears into the nose. The pressure on the eyeball from OOc contraction for forced closure of the eyes prevents the blood flow into the orbit vessels from becoming too violent. Pressure is thought to be exerted concomitantly on the lacrimal gland, thereby causing the excessive flow of tears often experienced at such times [4]. Skeletal muscle contractions including those of facial muscles therefore play important roles in physiological functions such as blood and tear flows. However, the positional relationships between the course of the angular vein and facial muscles have not been well described, nor has the possible role of those muscles as alternative venous valves. If the angular vein courses through sites where the facial muscles can efficiently compress it, the muscles can affect venous flow in the face, especially while facial expressions are performed.

Maes (1937) [12] suggested that the rich venous supply picks up infections in the face and rapidly carries them onward. This process is accelerated by both lip motions and the absence of valves in the facial vein. The angular vein, which might not have valves, can therefore be affected by facial muscle contractions in various postures and by bidirectional blood flow. The aim of this study was to elucidate the positional relationships between the courses of the angular vein and facial muscles, and to determine whether they can act as alternative venous valves.

## Materials and methods

The angular and facial veins of 44 specimens of embalmed adult Korean cadavers (10 males and 12 females) with a mean age of 72.1 years (range: 40–94 years) at the time of death were examined. The face was dissected bilaterally to expose the angular vein, facial vein, and facial muscles including the OOc, depressor supercilii (DS), zygomaticus minor (Zmi), Zmj, and levator labii superioris (LLS). The angular and facial veins were traced to observe their courses in relation to the muscles in the upper face, midface, and lower face. No history of trauma or surgical procedure was observed in any specimen.

This study was approved by the Institutional Review Board of the Catholic Kwandong University (IRB no. CKU-21-01-0409). All cadavers had been legally donated to the Catholic Kwandong University College of Medicine. Donors voluntarily consented to the dissection and preservation of their body for education and research purposes. The donor’s family agreed with the contents and procedures according to the will of the donors. None of the donors was from a vulnerable population and all donors or next of kin provided written informed consent that was freely given. The study was performed in accordance with the Declaration of Helsinki (64th WMA General Assembly in Fortaleza, Brazil in October 2013).

## Results

**Fig 1:**
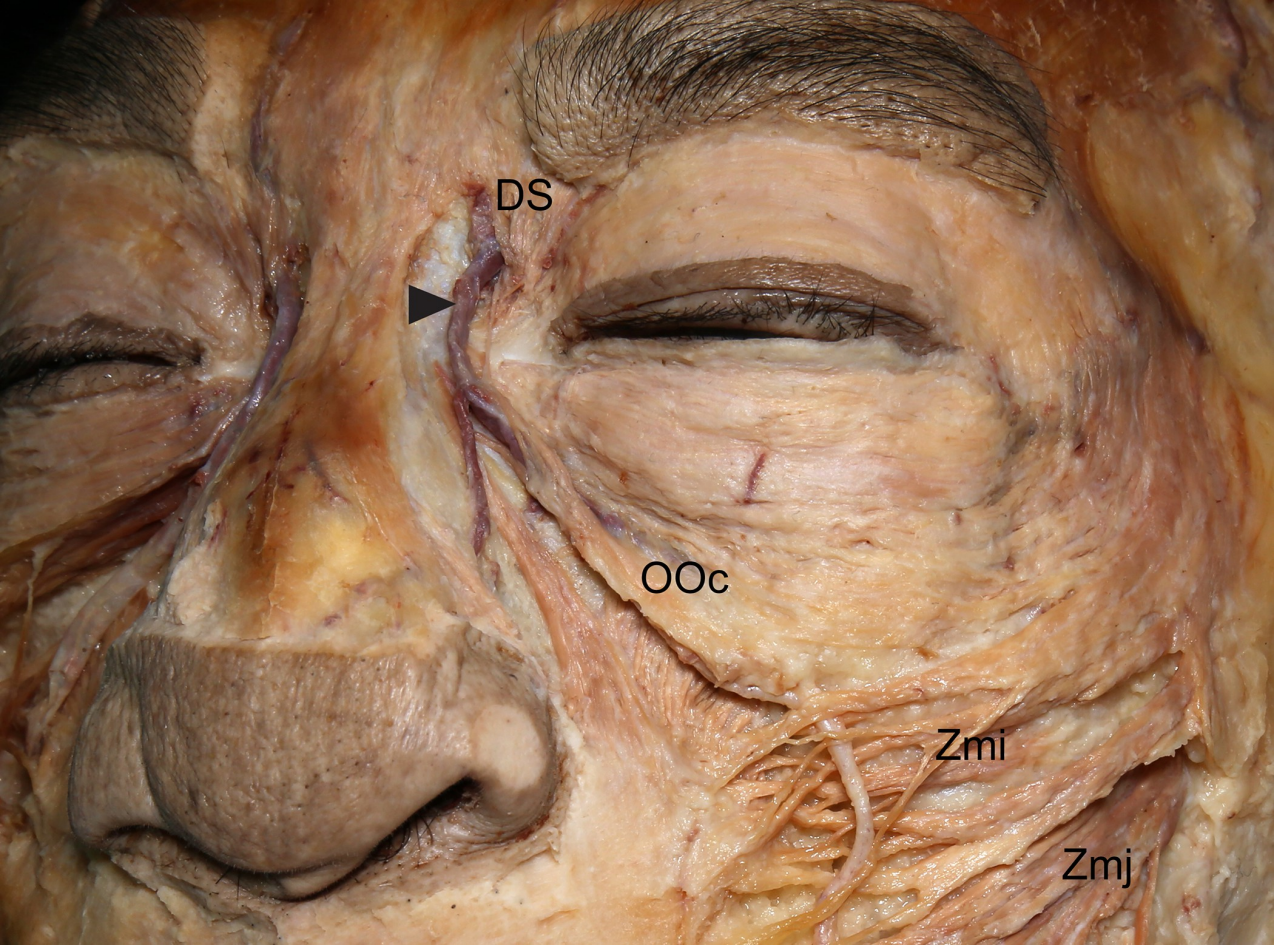
Course of the angular vein (arrowhead) in relation to the depressor supercilii (DS) in the upper face. The angular vein passed through the DS fibers and descended to the medial palpebral ligament. OOc, orbicularis oculi; Zmi, zygomaticus minor; Zmj, zygomaticus major.

**Fig 2:**
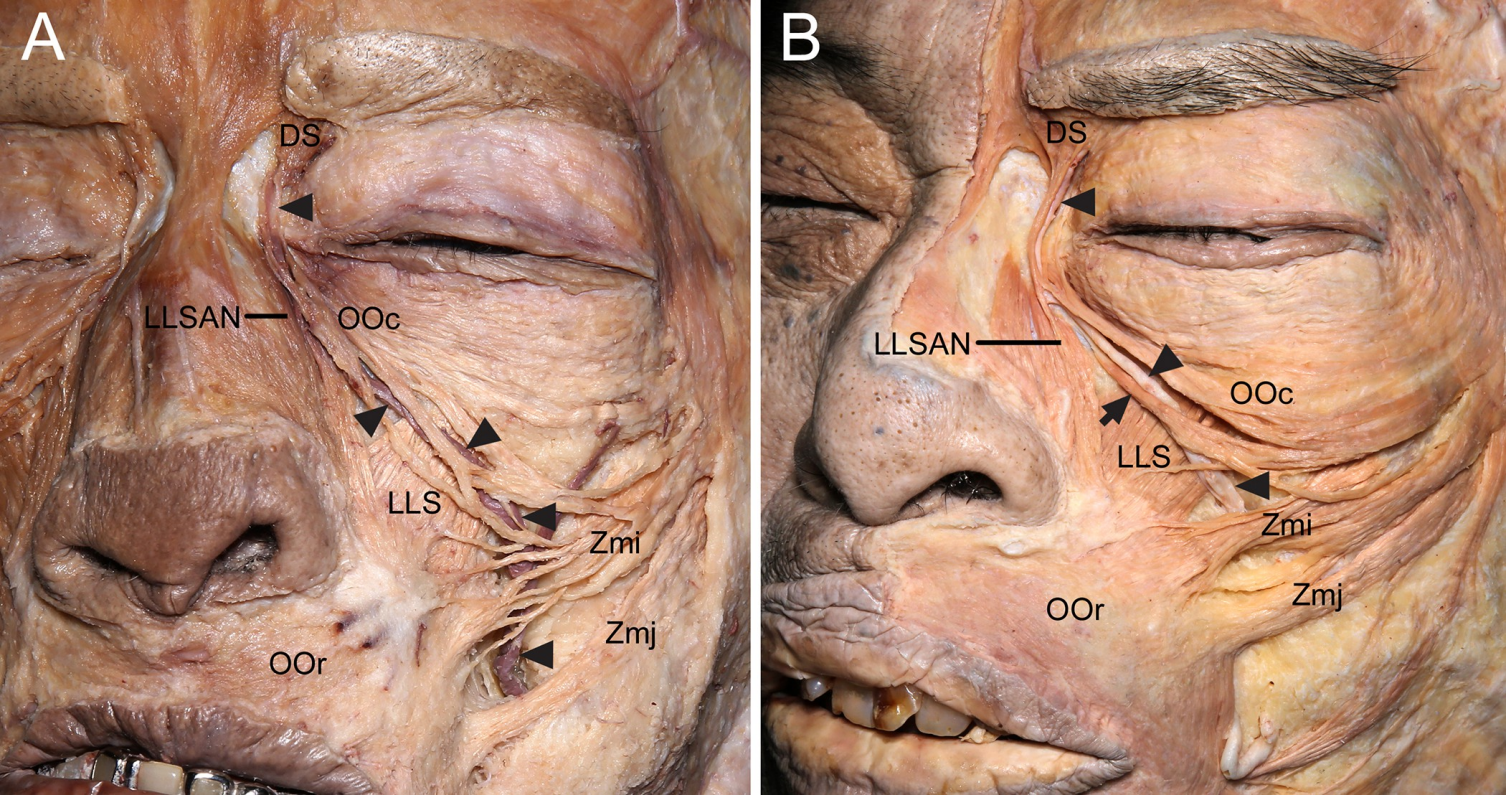
Course of the angular vein (arrowheads) in relation to the OOc in the midface. (A) The angular vein coursed along the deep surface of the inferior margin of the OOc. (B) When some inferior fibers (arrow) of the OOc were attached to the maxilla between the levator labii superioris alaeque nasi (LLSAN) and levator labii superioris (LLS), the angular vein coursed just above those fibers, and was covered by the inferior fibers of the OOc. The latter is slightly elevated to reveal the angular vein. OOr, orbicularis oris.

**Fig 3:**
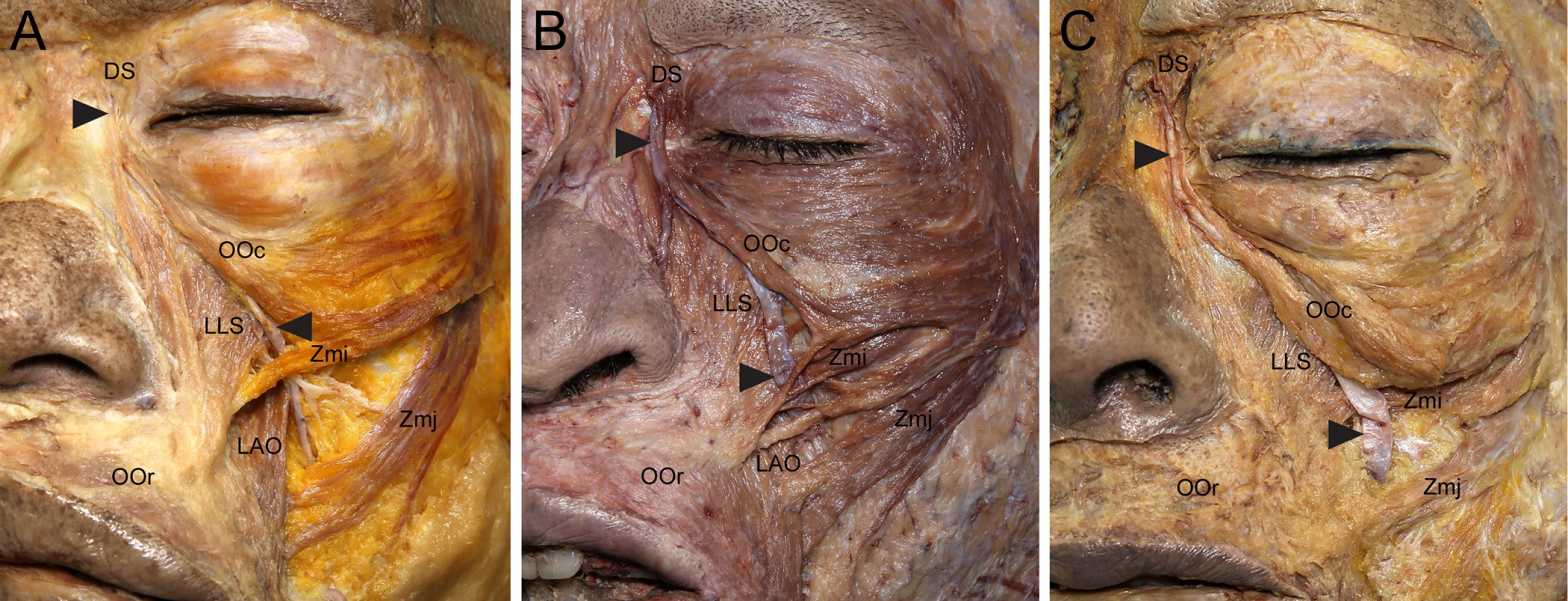
Course of the angular vein (arrowheads) in relation to the Zmi, LLS, and Zmj in the midface. At the level of the nasal ala, the angular vein passed between the LLS and Zmi (A), between the superficial and deep Zmi fibers (B), or between the Zmi and Zmj (C). LAO, levator anguli oris.

**Fig 4:**
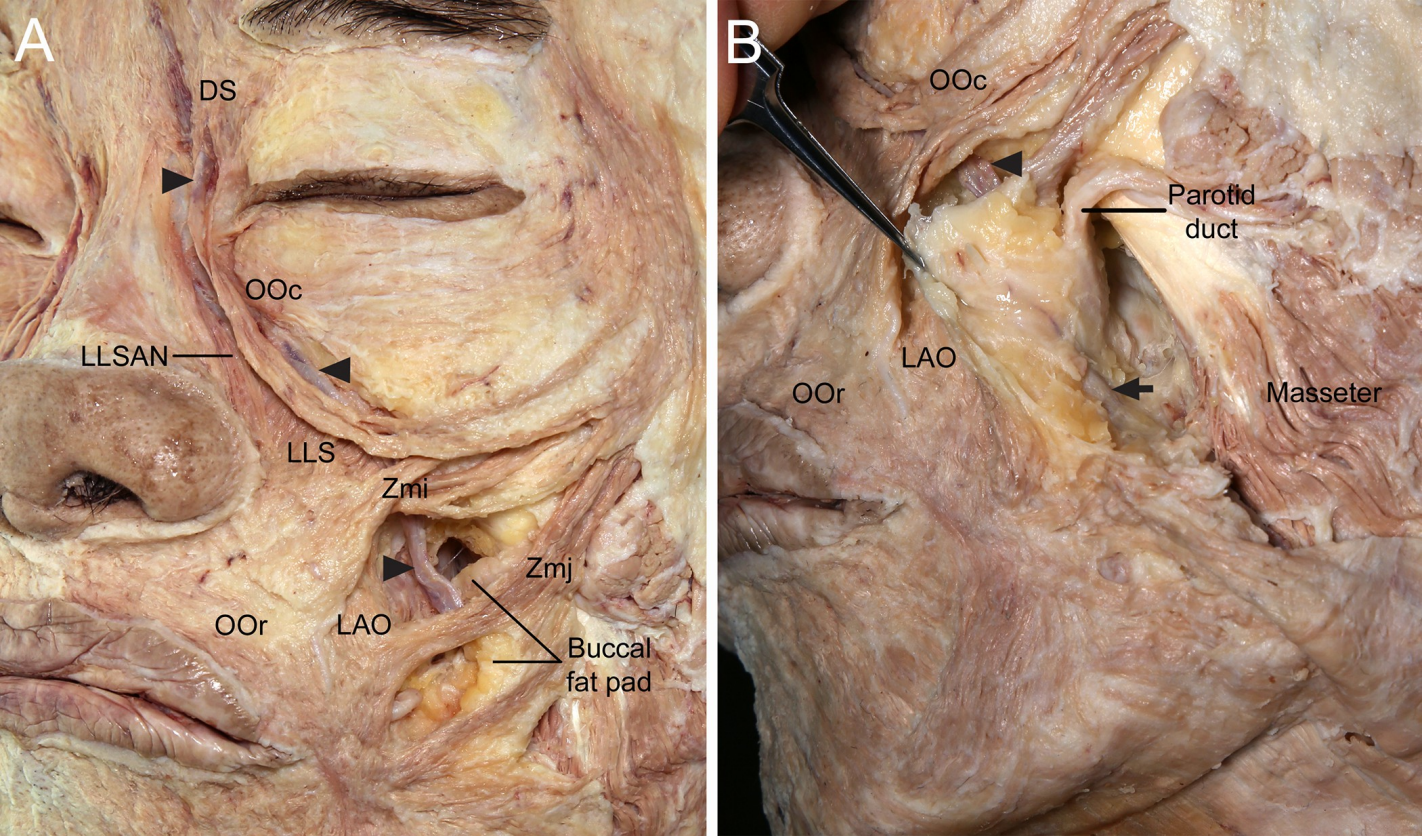
Course of the angular vein (arrowheads) in relation to the buccal fat pad in the lower face. (A) The angular vein coursed deep to the Zmj and lateral to the LAO. It passed through the anterior lobe of the buccal fat pad. Some of the buccal fat pad surrounding the angular or facial vein was removed to reveal those veins. (B) The anterior lobe of the buccal fat pad was reflected in the anterior view to show its posterior surface surrounding the angular or facial vein (arrow) and the parotid duct. The anterior lobe of the buccal fat pad was covered by the capsule, which divided it from the other lobes.

The course of the angular vein in the face was recorded in relation to the facial muscles and surrounding structures. In the upper face, it passed through the DS and descended to the medial palpebral ligament in all specimens (Fig 1). In the midface, it passed between the origin of the levator labii superioris alaeque nasi (LLSAN) and the inferior OOc fibers in all specimens. It coursed along the deep surface of the inferior margin of the OOc in all specimens (Fig 2A). In cases where some inferior OOc fibers attached to the maxilla between the LLSAN and LLS (6.8%, 3/44), the angular vein coursed just above those fibers, and was covered by the inferior OOc fibers (Fig 2B). It descended and crossed over the origin LLS fibers. At the level of the nasal ala, the course of the angular vein was classified into the following three types: in type I it passed between the LLS and Zmi (n = 17, 38.6%; Fig 3A), in type II it passed between the superficial and deep fibers of the Zmi (n = 21, 47.7%; Fig 3B), and in type III it passed between the Zmi and Zmj (n = 6, 13.6%; Fig 3C). In the lower face, the angular vein coursed deep to the distal Zmj fibers and lateral to the levator anguli oris in all specimens. After it joined the superior labial vein, it became the facial vein near the level of the upper lip. The angular or facial vein passed through the anterior lobe of the buccal fat pad in all specimens (Fig 4).

## Discussion

This study has revealed that the courses of the angular vein in the face appear to have close positional relationships with the facial muscles including the OOc, DS, and Zmi. As the angular vein courses deep to the margins of the facial muscles or passes between them, it can be compressed during muscle contractions, affecting venous flow and the spread of inflammation, especially under conditions of blood pooling such as supine, prone, or upside-down positions or during blood backflow during a Valsalva maneuver or exercise.

In the upper face, the angular vein passed through the DS fibers that depress the eyebrow, acting as a muscle of the glabellar complex. When the DS contracts, its fibers can compress the angular vein. Thus, blood flow from the angular to the superior ophthalmic vein and then to the cavernous sinus can be controlled, reducing the likelihood of inflammation spreading to the cavernous sinuses.

In the midface of all specimens, most parts of the angular vein coursed along the inferior margin of the OOc. The orbital portion of the OOc induces considerable lower eyelid elevation during eye closure [3]. The OOc is also important in facial expression and various ocular reflexes [3]. It is thought to be the main influencer of angular vein blood flow due to its frequent and forceful contractions and relatively large size.

The angular vein passed between the superficial and deep fibers of the Zmi, between the LLS and Zmi, or between the Zmi and Zmj. In the first two courses, it can be compressed more due to the narrow space between the muscle fibers. The Zmi also acts with the LLSAN and LLS as one of the muscles causing elevation during several facial expressions and mouth movements [3]. Contractions of the Zmi and LLS therefore compress the angular vein during frequent upper lip movements.

This study has revealed that the angular vein passes through the buccal fat pad in the lower face, where the mouth and mandible move frequently. Nishihara et al. (1995) [7] indicated that facial vein pressure greatly fluctuates in response to jaw movements. The buccal fat pad surrounding the angular or facial vein can therefore protect it from conflicts between facial and masticatory muscle contractions during mouth movements.

The experimental study of Cotofana et al. (2020) [13] found that smiling can significantly reduce venous flow in the angular or facial vein. Those authors also described that Zmj contraction compresses the vein against the underlying maxilla, and during both minimally invasive and surgical procedures that alter the superficial musculoaponeurotic system, the periocular musculature or the deep midfacial fat compartments might affect angular or facial venous flow. Another experimental study of Calomeni et al. (2022) [14] found that when the patients were asked to forcefully contract their OOc, the venous blood flow decreased from 10 cm/s to 7.3 cm/s, providing evidence that the OOc can alter the venous blood within the tear trough. The findings of these studies support our assumption that muscle contractions compress the vein, affecting venous flow and the spread of inflammation. Therefore, when surgical procedures are performed on structures that affect the facial muscles and angular or facial vein, particular care should be taken not to alter these structures.

The limitation of this study is that this observation based on the cadaveric dissection was unable to assess the blood flow of the angular vein. As a further study, venous blood flow and diameter of the angular vein or facial vein can be investigated via ultrasound imaging at the sites where the vein was located along with the muscles during several facial expressions.

## Conclusion

This study found that the angular vein coursed along the sites where facial muscle contractions are assumed to efficiently compress the veins, likely controlling venous flow as valves. The observations made and analysis performed in this study will improve the understanding of the physiological function of facial muscles as alternative venous valves.
